# A Review of Deep Learning Applications in Lung Ultrasound Imaging of COVID-19 Patients

**DOI:** 10.34133/2022/9780173

**Published:** 2022-02-15

**Authors:** Lingyi Zhao, Muyinatu A. Lediju Bell

**Affiliations:** 1 Department of Electrical and Computer Engineering, Johns Hopkins University, Baltimore, USA; 2 Department of Computer Science, Johns Hopkins University, Baltimore, USA; 3 Department of Biomedical Engineering, Johns Hopkins University, Baltimore, USA

## Abstract

The massive and continuous spread of COVID-19 has motivated researchers around the world to intensely explore, understand, and develop new techniques for diagnosis and treatment. Although lung ultrasound imaging is a less established approach when compared to other medical imaging modalities such as X-ray and CT, multiple studies have demonstrated its promise to diagnose COVID-19 patients. At the same time, many deep learning models have been built to improve the diagnostic efficiency of medical imaging. The integration of these initially parallel efforts has led multiple researchers to report deep learning applications in medical imaging of COVID-19 patients, most of which demonstrate the outstanding potential of deep learning to aid in the diagnosis of COVID-19. This invited review is focused on deep learning applications in lung ultrasound imaging of COVID-19 and provides a comprehensive overview of ultrasound systems utilized for data acquisition, associated datasets, deep learning models, and comparative performance.

## 1. Introduction

COVID-19 is a highly infectious disease caused by the novel SARS-CoV-2 virus, which was first identified in December 2019. In March 2020, COVID-19 was officially declared by the World Health Organization (WHO) as a pandemic [1]. With several similarities to the severe acute respiratory syndrome (SARS) and the Middle East respiratory syndrome (MERS) coronavirus diseases [2, 3], there have been more than 300 million reported cases of COVID-19 and over 5 million associated deaths worldwide [4]. The main symptoms of the disease include fever, dry cough, and shortness of breath [3]. Although infected patients can be asymptomatic or have mild symptoms and good prognoses [5], some cases can develop severe and even fatal respiratory diseases such as acute respiratory distress syndrome (ARDS) [5]. Considering the fast spread of COVID-19, quick and accurate diagnosis is both essential and urgent. Currently, the reverse transcriptase quantitative polymerase chain reaction (RT-qPCR) test is considered as a gold standard for diagnosing COVID-19 [6]. Although the test is overall deemed accurate [6], it is time-consuming and may take more than 24 hours to obtain results. In addition, the requirement of bio-molecular testing facilities limits its availability in large scales and less developing regions. Alternatives to RT-qPCR tests include imaging techniques such as chest computed tomography (CT) [7], chest X-ray (CXR) [8], and lung ultrasound (LUS) [9, 10], which have each shown potential for the diagnosis of the COVID-19.

Chest CT has been recommended for hospitalized, symptomatic COVID-19 patients with specific clinical indications [11]. The most observable CT features discovered in COVID-19 pneumonia include bilateral, peripheral, and basal predominant ground-glass opacities and/or consolidations [12]. One limitation of CT is that it requires patient relocation because most fever clinics are relatively simple and do not include CT equipment. Moreover, to decrease the contagion risk for physicians and other patients, disinfection is essential after each examination [13]. CXR, on the other hand, is a more preferred first-line imaging modality with lower cost and a wider availability for detecting chest pathology. Some of the CXR results of COVID-19 patients showed consolidation [8]. However, a large-scale study showed that for 636 CXRs from COVID-19 patients, 58.3% were reread as normal, and 41.7% were reread as abnormal [14]. With the relatively low sensitivity of CXR, the American College of Radiology (ACR) recommends performing CXR with portable units in ambulatory care facilities only if medically necessary [11].

**Figure 1:**
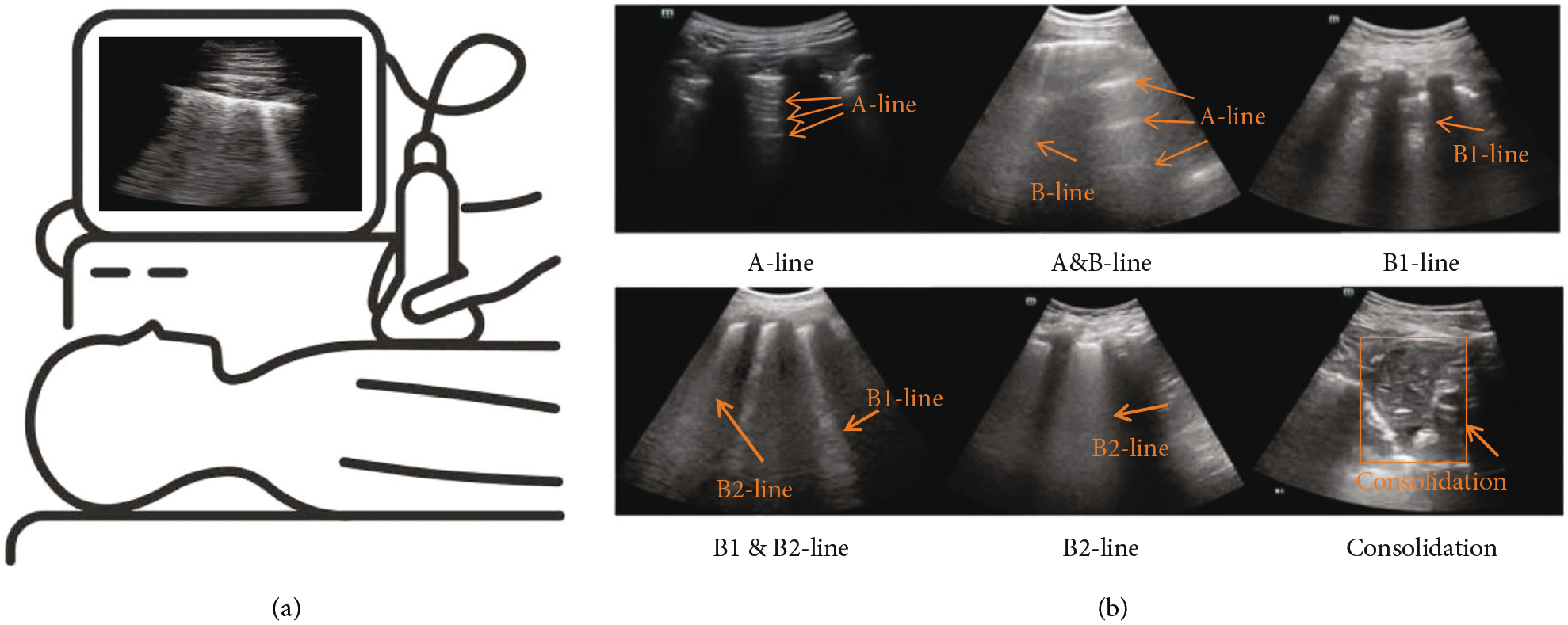
(a) Illustration of lung ultrasound imaging. (b) Common ultrasound image features appearing in lung examinations (modified material from Hu et al. [25]; licensed under CC BY 4.0 [26]).

Compared with CT and X-ray, ultrasound does not produce ionizing radiation, is more cost-effective, and has better diagnostic accuracy to detect pleural effusions, interstitial syndrome, alveolar-interstitial disorders, and consolidations, when compared to CT [15–17]. In addition, due to the portability of ultrasound devices, LUS does not require relocating the patient and thus can minimize the potential risk of further infection. Over the past year, LUS has been useful for the evaluation of acute chronic conditions including cardiogenic pulmonary edema, acute lung injury, pneumonia, and many other lung diseases [10, 18]. Figure 1 illustrates four common features for detection of these diseases in LUS. The A-line is a horizontal reverberation artifact of pleura caused by multiple reflections, representing a normal lung surface [19], because a healthy lung mainly consists of air. Ultrasound waves are thus reflected by the visceral pleural plane, typically causing acoustic reverberations between the pleural plane and skin surface, resulting in the appearance of A-lines. B-lines, also known as B1-lines, are denoted by a discrete laser-like vertical hyperechoic artifact that spreads to the end of the screen [20], representing the inter-lobular septum. B-lines occur because the pleural plane is no longer a specular reflector when the ratio between air, tissue, fluid, or other biological components is reduced. Consequently, various types of localized B-lines extending from the pleural plane appear [21–23], representing alterations of the subpleural tissue [21, 22]. A fusion B-line, also called a B2-line, is a sign of pulmonary interstitial syndrome, which shows a large area filled with B-lines in the intercostal space [20]. Finally, a pulmonary consolidation is characterized by a liver-like echo structure of the lung parenchyma, with a thickness of at least 15 mm [24].

For COVID-19, the most common abnormality is interstitial involvement depicted as B-pattern (i.e., three or more B-lines present in a lung region, confluent B-lines, or white lung appearance) [27]. LUS patterns are also reported to be correlated with disease stage, comorbidities, and severity of pulmonary injury [28], suggesting its potential for long-term monitoring. Although LUS has shown great potential in the evaluation of COVID-19, it is not mentioned in the ACR recommendations as clinical practice for COVID-19 [11]. Possible reasons include highly variable operator dependence when using LUS equipment and interpreting LUS images, and standardized protocols for LUS imaging of COVID-19 are not yet established.

As a powerful tool for predictions and interpretability assistance, artificial intelligence (AI) has gained much interest in healthcare. AI applications in healthcare include disease detection, treatment selection, patient monitoring, and drug discovery [29]. As a subset of AI techniques, deep neural networks have quickly permeated medical imaging applications. These applications include image registration, detection of anatomical and cellular structures, tissue segmentation, computer-aided disease diagnosis, and prognosis [30]. For ultrasound imaging, in particular, deep learning has rapidly gained recent attention in several aspects [31], ranging from beamforming [32–34] and compressive sampling [35] to speckle suppression [32, 36], segmentation [32, 37], and automated or radiologist-assisted disease assessment [38–42]. While promising deep learning applications for diagnostic ultrasound B-mode imaging rely on the identification of physical structures within organs such as the breast [38–40], liver [41], prostate [37], and kidney [42], deep learning applications for ultrasound imaging of the lungs primarily rely on the presence of image artifacts (e.g., acoustic reverberations that appear as A-lines or B-lines). In addition, while multiple research groups have proposed deep learning for the diagnosis of COVID-19 based on defined structures in CT and X-ray images, fewer studies have reported using deep learning to diagnose COVID-19 with LUS [43].

**Figure 2:**
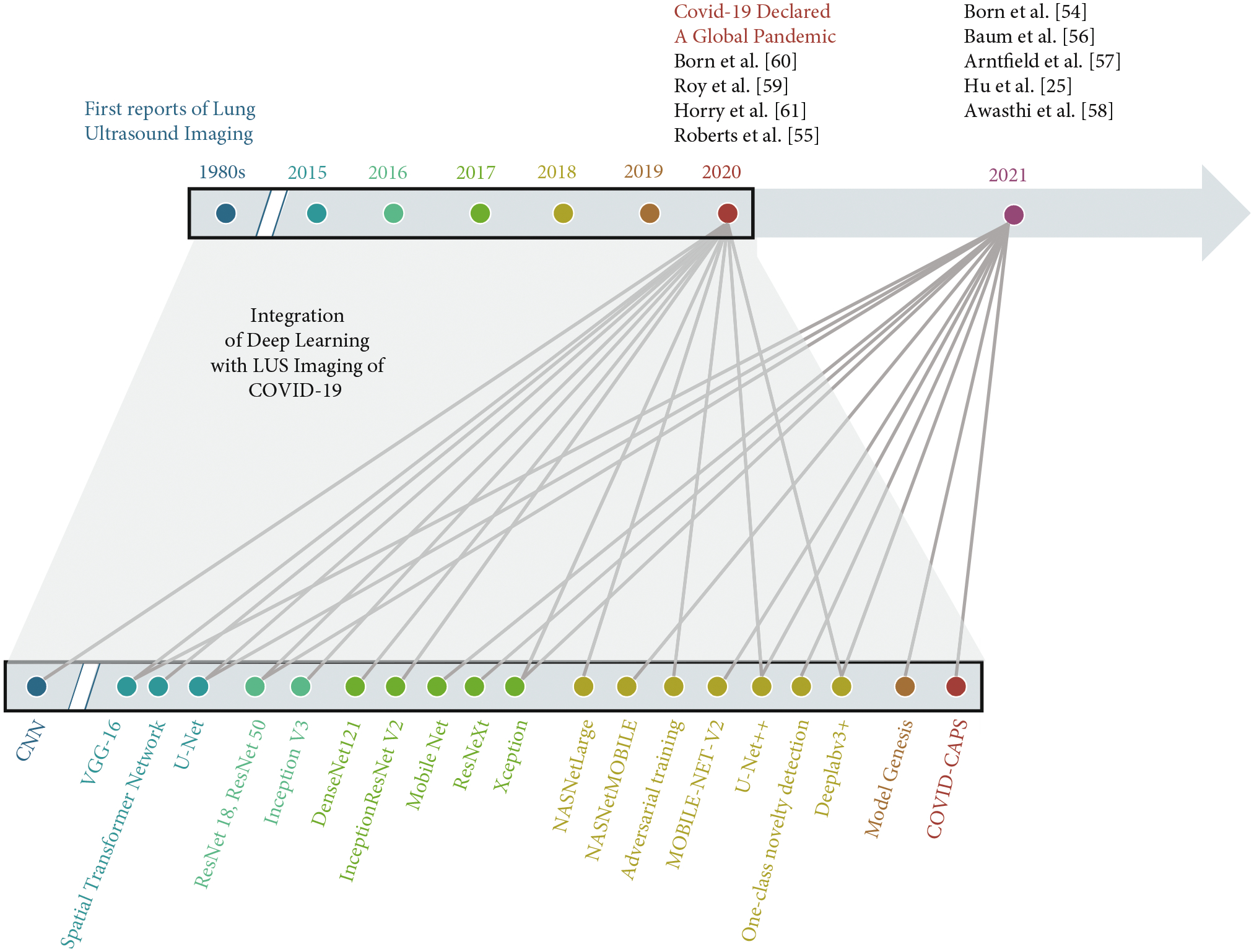
Timeline illustrating the integration of lung ultrasound imaging with deep learning to achieve COVID-19 detection. Gray lines link the publication years of the articles summarized herein to the deep learning architectures utilized in each article, color coded by publication year.

Our objective in this review is to draw more focused attention to LUS approaches that utilize deep learning techniques to diagnose COVID-19. We review a total of nine articles using fully supervised approaches primarily applied to patients with COVID-19. The first reports of LUS imaging of the features in Figure 1 appeared in the 1980s [44–47] and paved the way for the nine reviewed articles appearing approximately 40 years later, as summarized at the top of Figure 2. This timeline is juxtaposed with and mapped to an exploded timeline view of the deep learning architectures utilized in these reviewed articles, wherein the first convolutional neural network (CNN) was introduced in the 1980s [48–51] (similar to the first reports of LUS imaging features of interest). We limit our review to network inputs containing three or less channels, and we omit fusion approaches (e.g., [52, 53]) to maintain a focus on comparable approaches. The nine reviewed articles appeared in print from May 2020 to March 2021 and provided the research community with initial expectations for success when integrating deep learning with LUS imaging of COVID-19. A summary of the number of training, testing, and validation examples used in each study appears in Table 1, with additional details about the datasets and data sources for each of the studies available in Table 2.

The remainder of this article is organized as follows: Section 2 discusses four manuscripts containing explainable deep learning applications, while the remaining studies in this review apply deep learning in LUS imaging of COVID-19 patients without an explainability analysis. Section 3 discusses new deep learning architectures exclusively developed for COVID-19 detection. Section 4 discusses open-access resources for deep learning in LUS analysis of COVID-19 patients. Section 5 compares LUS deep learning outcomes with results from other medical imaging techniques. Finally, Section 6 concludes the manuscript with a summary and outlook. Overall, we anticipate that readers will gain: (1) an overview of initial deep learning approaches integrating deep learning and LUS; (2) a summary of ultrasound imaging systems, data, and networks that made these initial applications possible; and (3) an understanding of the promise of this research area, existing gaps, and associated room for improvement and growth.

## 2. Explainable Deep Learning Applications

While the validity of explaining deep learning results has been debated [65], the existence of this approach nonetheless persists, and there are five articles applying explainable deep learning architectures in LUS imaging of COVID-19 patients [54–58]. First, Born et al. [54] released the largest publicly-available LUS dataset (202 videos + 59 images), comprising samples of COVID-19 patients, patients with bacterial pneumonia, (non-COVID-19) viral pneumonia, and healthy controls. In addition to clinical data donated from hospitals, published in multiple open repositories, the dataset also included clinical data collected by the authors themselves in two healthcare organizations using a Venue ^™^ ultrasound machine (GE Healthcare, Ltd., IL, USA). Both convex and linear array ultrasound probes were used to acquire these data. Several frame-based convolutional neural networks as well as video-based convolutional neural networks for classifying COVID-19, pneumonia, and healthy patients were then compared. Networks were trained on 1,204 images from COVID-19 patients, 704 images from patients with bacterial pneumonia, and 1,326 images from healthy individuals. These images were released in a public database, compiled from 179 videos and 53 images total [54].

Born et al. [54] investigated both frame- and video-based classification. For frame-based classification, Born et al. [54] compared NaNET Mobile [66], VGG-Segment, and Segment-Enc with two VGG-16 based architectures named VGG and VGG-CAM. NaNET mobile [66] is a lightweight neural network that uses less than 1/3 of the parameters of VGG-16 and was optimized for applications on portable devices. VGG-segment and Segment-Enc were two approaches built upon the pretrained model of an ensemble of three U-Net-based models (U-Net, U-Net++, and DeepLabv3+) [59]. VGG-segment was identical to VGG but was trained on the segmented images from the ensemble. In Segment-Enc, the bottleneck layer of each U-Net-based model was used as a feature encoding of the images and was fed through a two-layer multilayer perception. VGG-CAM enabled the usage of class activation maps (CAMs). A CAM indicated the discriminative image regions used by the convolutional neural network (CNN) to identify a given category [67]. Both VGG and VGG-CAM achieved similarly promising performance with an accuracy of 88 ± 5*%* on a 5-fold cross-validation of 3,234 frames, where the accuracy is the proportion of cases correctly identified as COVID-19, healthy, or pneumonia (see details in Table 3). For video-based classification, in addition to selecting the class with the highest average probability obtained by the frame-based classifier VGG-CAM, Born et al. [54] also investigated Model Genesis [68]. The VGG-CAM based classifier outper-formed Model Genesis, producing a video accuracy of 90% compared to the 78% accuracy obtained with Model Genesis.

**Figure 3:**
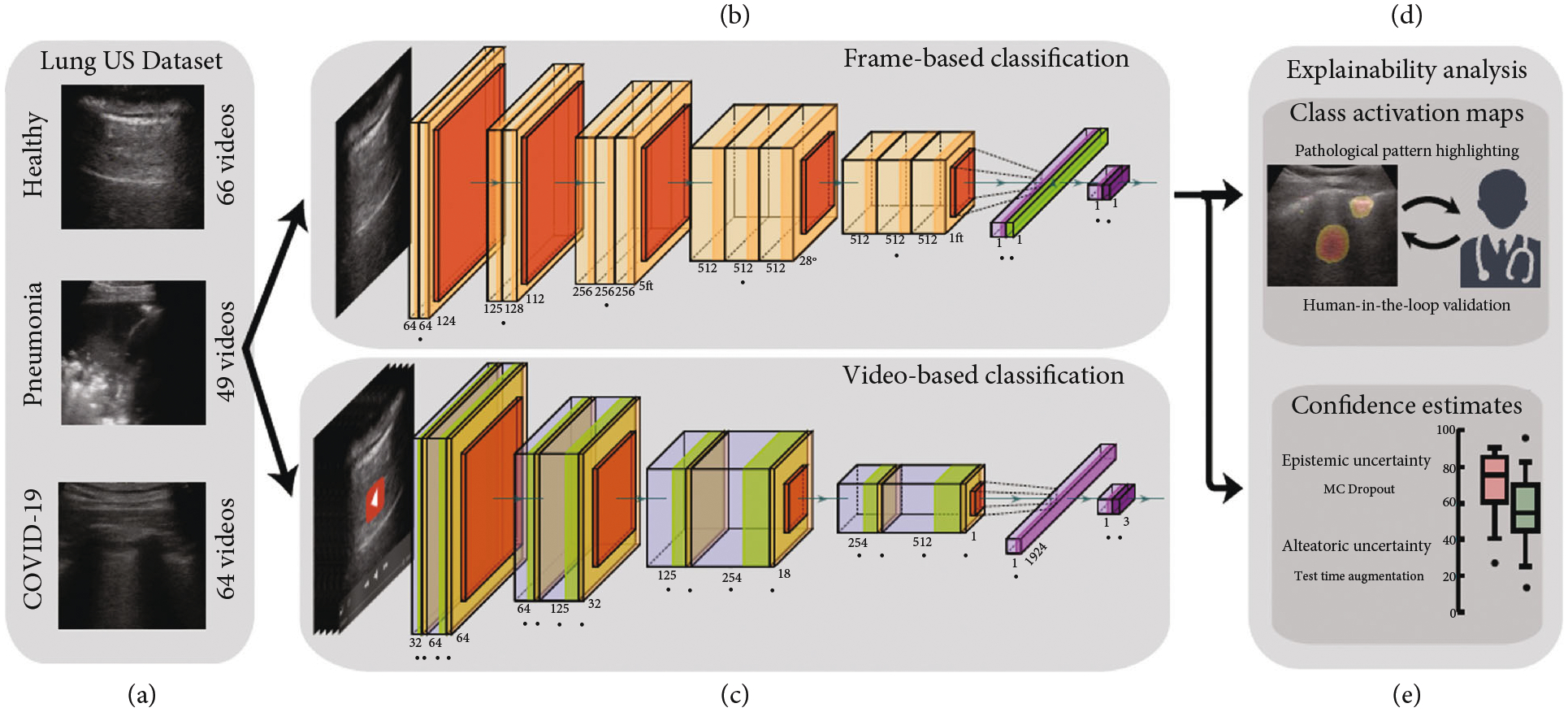
Flow chart of the method proposed by Born et al. [54], including (a) 3 examples from the LUS dataset, (b) frame-based and (c) video-based CNN fine-tuned on the LUS dataset, (d) class activation maps that highlight patterns driving the decision of the model, which were then reviewed and evaluated for diagnostic value by medical experts, and (e) uncertainty techniques are employed and shown to equip the model with the ability to recognize samples with high error probability. Modified material from [54]; licensed under CC BY 4.0 [26].

To explain performance, Born et al. [54] employed CAM techniques [67] and confidence estimates, using the work-flow shown in Figure 3. To investigate the explanatory power of the CAMs, two medical experts experienced in the ultrasound diagnostic process were asked to score activation maps for 50 correctly classified videos on a scale of −3 (indicating “the heatmap is only distracting”) to 3 (indicating “the heatmap is very helpful for diagnosis”). The CAMs were overall perceived useful and scored best for videos of bacterial pneumonia. When considering confidence estimates, the epistemic confidence estimate was found to be highly correlated with the correctness of the predictions while the aleatoric confidence was found correlated to a lesser extent.

Roberts and Tsiligkaridis [55] presented work exploring the robustness of using deep CNNs to make COVID-19 diagnostic decisions with LUS by applying adversarial training. Adversarial training is an effective defense against adversarial attacks to which traditional neural networks are vulnerable [69]. In addition, according to [70], adversarial attacks can also be used to discern features that a model has learned. For models with adversarial training, these features have shown to be better aligned with human perception than the models without adversarial training. To find the features the model has learned, Roberts and Tsiligkaridis [55] considered a framework based on the work of Tsiligkaridis and Roberts [71]. This approach finds pertinent negatives (i.e., misclassified features) and pertinent positives (i.e., critical features that are present in the input examples) by optimizing over the perturbation variable *δ*.

For the training process, two networks—VGG 16 [72] and ResNet18 [73]—were trained on the updated POCOVID dataset [60], which included 3,119 frames from 195 ultrasound videos. For each network, both standard training and adversarial training were performed. Results demonstrated that the models with adversarial training (named robust models) have less sensitivity than the models with standard training (named standard models). Specifically, the VGG16-robust model achieved an accuracy of 81.498% for COVID-19, which was lower than that achieved from the VGG16-standard model, which was 85.992%. Here, the accuracy is defined as the proportion of cases correctly identified as COVID-19 or non-COVID-19 (including healthy and pneumonia cases), with more definition details available in Table 3. When applying increasingly strong adversarial attacks, the performance of the standard models degraded compared to the robust models, suggesting that the standard models learned features that were sensitive to idiosyncrasies or noise in the training dataset. In general, the perturbations of robust models were more focused and medically relevant than the perturbations of the standard models, which were diffuse and less interpretable. The interpretation of these perturbations is that the standard model seems to only focus on the brighter parts of the image, while the robust models seem to focus on more distinct features of the original image.

Baum et al. [56] proposed to add a quality assessment module before the diagnostic classification module, with guided gradient-weighted CAMs [59, 74] calculated to illustrate regions of interest in classification, also known as Grad-CAMs. For quality assessment, Baum et al. [56] compared three modules. The first model was a binary classification network (QA^bin^) based on VGG [72]. Training QA^bin^ required manual labeling of the data as having either sufficient or insufficient quality. The second model was an adversarial deep learning model capable of novelty detection (QA^nd^) [75, 76], which required only COVID-19-positive examples. The third quality assessment method QA^bin+nd^ combined QA^bin^ and QA^nd^, using a Bayesian model. The quality assessment module was followed by a diagnostic classification module *D*^bin^.

The datasets used for training and testing were obtained in two hospitals in the UK. In total, 25,800 LUS images were acquired from 37 COVID-19 positive patients, and 16,627 images were acquired from 12 COVID-19 negative cases. A Butterfly iQ ultrasound probe (Butterfly Inc., Guilford, CT, USA) was used to obtain the patient images. A total of 937 images were annotated as insufficient quality by an experienced ultrasound imaging researcher. The proposed quality assessment networks, QA^bin^, QA^nd^, and QA^bin+nd^ were trained on data from one hospital. The diagnostic classification network *D*^bin^ was trained with five-fold cross-validation on data from the second hospital. Before diagnostic classification, each fold of data was evaluated independently by QA^bin^, QA^nd^, and QA^bin+nd^.

**Figure 4:**
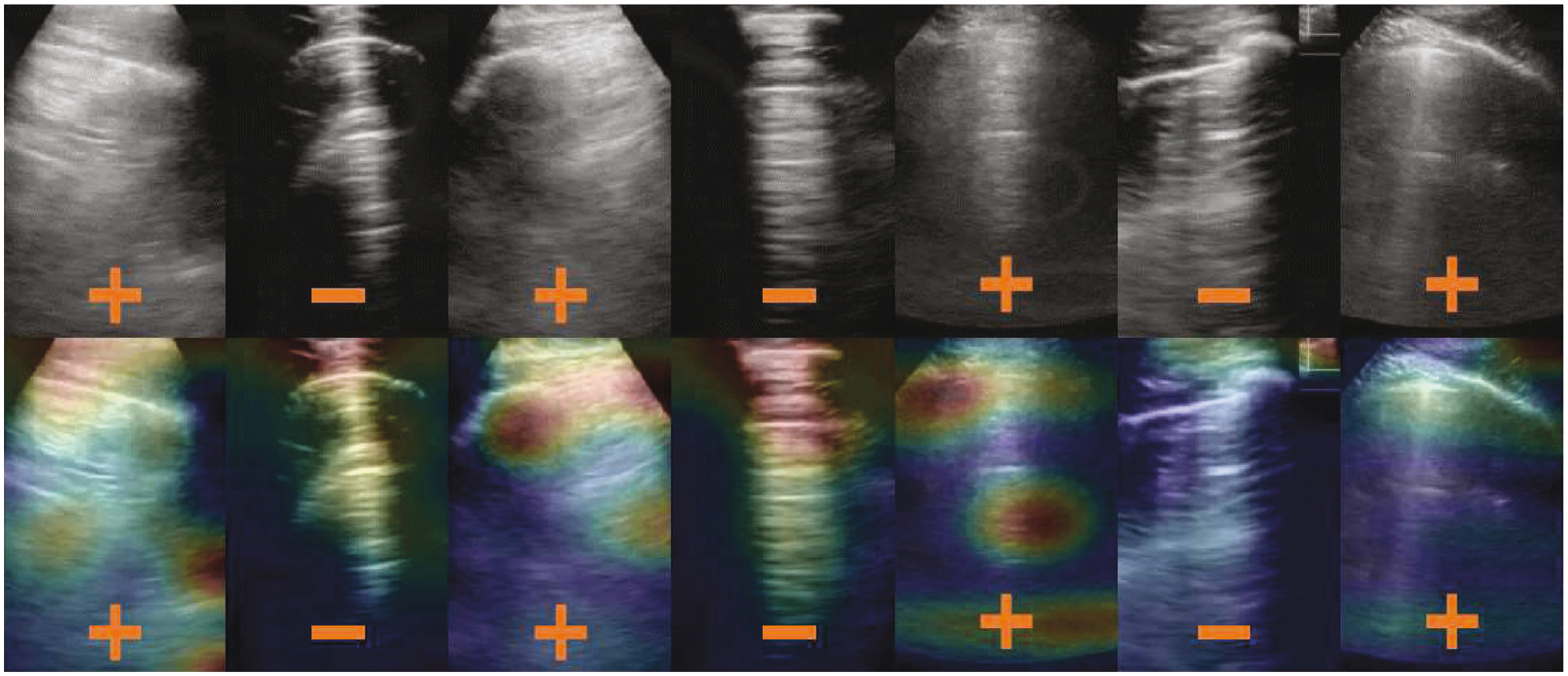
Example results from Baum et al. [56]. The + and – signs indicate true-positive and true-negative COVID-19 diagnoses, respectively. LUS images in the top row are overlaid with guided gradient-weighted class activation maps (Grad-CAMs) in the bottom row. ©2021 SPIE. Reprinted, with permission, from [56].

The resulting quality assessment demonstrated that the classification was 0.85 when using QA^bin^ or QA^nd^ alone and was 0.86 when using QA^bin+nd^. The classification accuracy of *D*^bin^ without any quality assessment was 0.95. After rejecting images of insufficient quality with quality assessment module QA^bin^, QA^nd^, and QA^bin+nd^, the classification accuracies of *D*^bin^ were 0.95, 0.97, and 0.95, respectively. The authors suggested that when training with more data of insufficient quality, the improvements are likely to be larger and will be more impactful for less experienced users. The guided gradient-weighted class activation maps (Grad-CAMs) shown in Figure 4 indicate that the networks have learned meaningful, human interpretable LUS features.

Arntfield et al. [57] explored whether deep learning models can match or exceed humans in the diagnosis of COVID-19 with LUS images of similar pathological appearance. The exams were performed at two Canadian tertiary hospitals of London Healthy Sciences Centre. A variety of ultrasound systems were used for data collection, including Edge, X-porte, Edge-2, S-Cath ultrasound systems by Sonosite (FUJIFILM Sonosite, Inc., WA, USA), a Lumify ultrasound system by Philips (Philips Medical Systems, Inc., the Netherlands), and an M9 ultrasound system by Mindray (Mindray Bio-Medical Electronics Co., Ltd., China). Phased array ultrasound probes were predominantly used for these data acquisitions. In total, 612 LUS videos of B-lines from 243 patients with either COVID-19 (*n* = 84), non-COVID acute respiratory distress syndrome (NCOVID, *n* = 78), or hydrostatic pulmonary edema (HPE, *n* = 81) were included in this study.

In choosing an optimal training architecture for classification, Arntfield et al. [57] investigated training on CNNs and residual CNNs, as well as transfer learning methods. The performance of each model was assessed by calculating the area under the receiver operating characteristic curve (AUC) and analyzing the confusion matrix. The results were analyzed at both the frame level and the patient level. To visually explain the model’s predictions, the Grad-CAM method was applied. For comparison of human performance and model performance, a survey including 25 lung ultrasound videos was distributed to 100 LUS-trained acute care physicians from across Canada. Respondents were asked to identify the cause of the LUS findings (HPE, non-COVID, or COVID).

Among the seven common architectures evaluated, Xception performed best in distinguishing between the three relevant causes of B-lines with AUCs of 1.0 (COVID), 0.934 (non-COVID), and 1.0 (HPE) at the patient level, resulting in an overall AUC of 0.978. The AUCs obtained from the physicians, on the other hand, were 0.697 (COVID), 0.704 (non-COVID), and 0.967 (HPE), producing an overall AUC of 0.789, far less than the overall AUC achieved from the classification model. Furthermore, the confusion matrix obtained from the physicians showed a near-random classification between COVID and non-COVID, suggesting that distinguishing between these two classes is hardly possible for humans. Visualizations with Grad-CAM indicated that the key activation areas for all classes investigated were centered around the pleura and the pleural line. Heat map visualizations also highlighted image variations that were not obvious, yet were thought to contribute to the overall performance of the model.

Awasthi et al. [58] developed a lightweight, mobile-friendly, efficient deep learning model for detection of COVID-19 using LUS images. The proposed model, Mini-COVIDNet, was a modified MobileNet model, which utilized depthwise separable convolutions and pointwise convolutions for a reduction in size [77]. To improve model performance on an imblanced ultrasound dataset, Mini-COVIDNet employs focal loss [78, 79], rather than the entropy loss that is otherwise utilized in the MobileNet model.

Mini-COVIDNet was compared with five alternative deep learning models: (1) COVID-CAPS, which was previously utilized to identify COVID-19 infected cases in CXR images [80]; (2) POCOVID-Net, which is described in Section 4 [60]; (3) ResNet, a convolution part of ResNet50 [73], which is known to provide good performance on very large computer vision datasets set such as ImageNet; (4) MOBILE-Net-V2, a modified version of MobileNet previously shown to improve performance among other lightweight deep learning models [81]; and (5) NASNetMOBILE, which utilizes a new search space to provide more generalizability of the model for better performance in classification tasks [66]. These models were implemented with and without focal loss for comparison. A scaled version of COVID-CAPS was additionally implemented to match the number of parameters in Mini-COVIDNet.

Each model performance was evaluated by reporting sensitivity, specificity, precision, and *F*1-score (see Table 3 for definitions) for three classes (i.e., COVID-19, healthy, and pneumonia). In addition, the accuracy for three classes combined was also reported. Among the compared models, the memory size requirement of COVID-CAPS was the lowest, yet precision and *F*1-score were poor compared to other models. POCOVID-Net performance was similar to Mini-COVIDNet while the number of parameters in Mini-COVIDNet was smaller by a factor of 4.39. In addition, Mini-COVIDNet also required less memory and less training time than POCOVID-Net.

**Figure 5:**
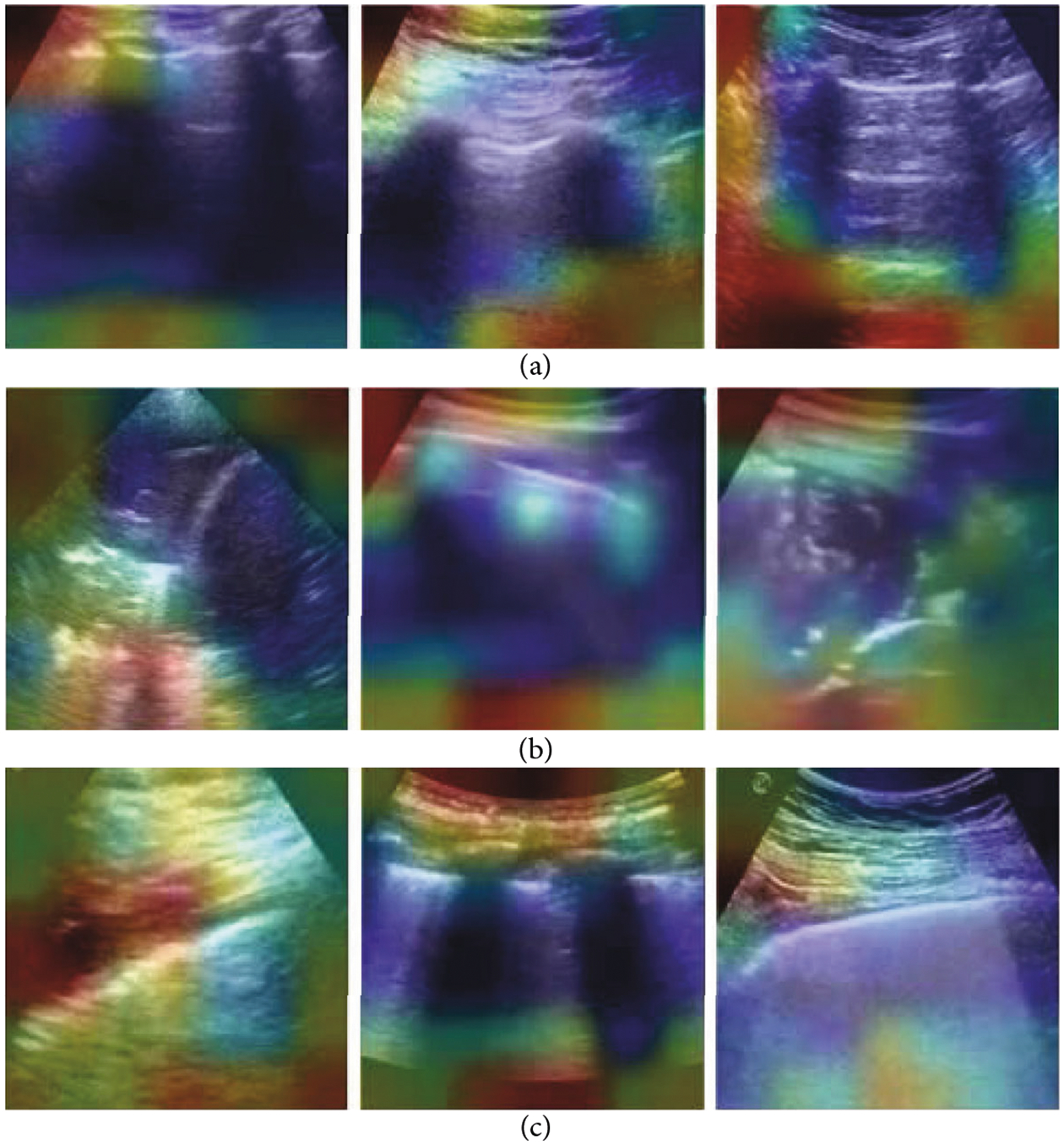
Example Grad-CAM visualizations of Mini-COVIDNet applied to LUS images of (a) healthy lungs, (b) pneumonia infected lungs, and (c) COVID-19 infected lungs. ©2021 IEEE. Reprinted, with permission, from Awasthi et al. [58].

Mini-COVIDNet employing focal loss [78, 79] provided a sensitivity of 0.92, a specificity of 0.71, a precision of 0.83, and an *F*1-score of 0.87 when differentiating the COVID-19 class from the non-COVID-19 classes (including pneumonia and healthy cases), as well as an accuracy of 0.832 for the three classes combined. In terms of memory size and training speed, Mini-COVIDNet required minimal memory (i.e., 51.29 MB) and less than 30 minutes training time. As shown in Figure 5, Grad-CAM visualization of learned features with Mini-COVIDNet highlighted the pleural line and A-line features in healthy lungs, pleural consolidations in pneumonia cases, and irregular pleural lines with B-line artifacts in COVID-19 cases. It is also important to note that these general explainability characterizations were not consistently present across all cases.

## 3. New Architectures for COVID-19 Detection

Rather than relying on “out-of-the-box” deep learning architectures, three studies [25, 56, 59] proposed new architectures in deep learning applications of LUS imaging of COVID-19 patients, including the architecture by Baum et al. [56] discussed in the preceding section. The remaining two new architectures are discussed in this section.

**Figure 6:**
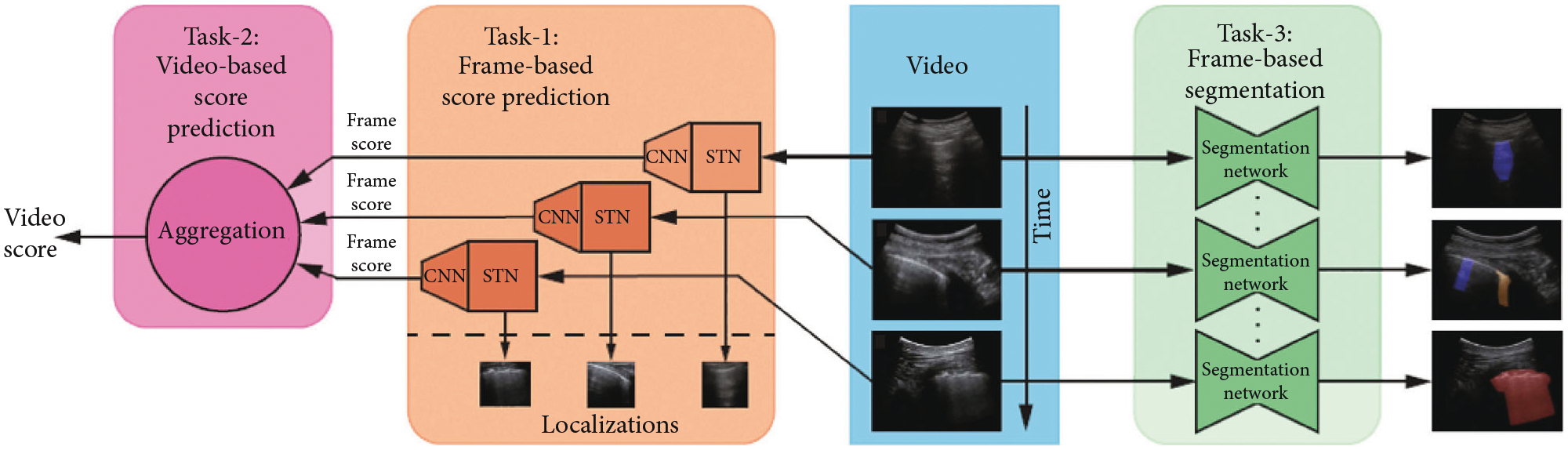
Overview of the deep learning architecture developed by Roy et al. [59] to achieve multiple tasks. Task 1: prediction of the disease severity score for each input frame and weakly supervised localization of pathological patterns. Task 2: aggregation of frame-level scores for producing predictions on videos. Task 3: estimation of segmentation masks indicating pathological artifacts. ©2020 IEEE. Reprinted, with permission, from [59].

Roy et al. [59] presented the Italian COVID-19 Lung Ultrasound DataBase (ICLUS-DB), which included 277 lung ultrasound videos (58,924 frames) from 35 patients (17 COVID-19, 4 COVID-19 suspected, and 14 healthy). The data were acquired within 5 clinical centers in Italy with both linear and convex ultrasound probes. A variety of ultrasound scanners were used to acquire these data, including DC-70 Exp (Mindray Bio-Medical Electronics Co., Ltd., China), MyLabAlpha (Esaote, Italy), Aplio XV (Toshiba, Ltd, Japan), and WiFi Ultrasound Probes (ATL, Italy). Each image in the dataset was annotated with the degree of the progression of the pathology (score 0 to 3) based on the scoring system devised previously by the same group [82]. Video-level annotations of a subset of 60 videos sampled across all 35 patients were also obtained. In addition, 1,431 frames from 33 patients were semantically annotated at a pixel level by contouring the corresponding regions. Figure 6 shows the overview of different tasks considered in this work.

For frame-wise score prediction, Roy et al. [59] introduced a novel deep architecture, displayed in Figure 6 which leveraged Spatial Transformers Network (STN) [83] and consistency losses [84] to localize disease patterns. To make the prediction more robust, Roy et al. [59] proposed Regularised Spatial Transformer Networks (Reg-STN). The regions localized by Reg-STN were then provided to a CNN [85] for classification. Soft ordinal regression (SORD) [86] was used in the loss function because labels were annotated from an ordinal scale.

To estimate video scores, Roy et al. [59] introduced a lightweight approach based on uninorms [87, 88]. The proposed uninorm-based aggregation was compared with two standard aggregation methods: max_argmax and argmax_ mean. For semantic segmentation, three models including U-Net [89], U-Net++ [90], and DeepLabv3+ [91] were compared. To further improve robustness and performance, Roy et al. [59] applied ensemble learning by calculating the unweighted average over prediction scores provided by the U-net, U-net++, and DeepLabv3+.

The results in [59] show that for frame-based score predictions, the proposed network achieved an *F*1 score of 65.1 on the test set, the highest among all compared networks (see details in Table 3). For video-based score prediction, the proposed uninorms aggregation method achieved the highest weighted *F*1 score, precision, and recall of 61 ± 12 *%*, 70 ± 19*%*, and 60 ± 7*%*, respectively. For semantic segmentation, the results demonstrate that the ensemble model yielded the most substantial performance gain over a baseline U-Net, increasing the Dice coefficient from 0.64 to 0.75 for the union of COVID-19 markers.

Hu et al. [25] proposed a new classification network for the fully automatic assessment of lung involvement in COVID-19 patients using three datasets collected in four Chinese medical centers. The three ultrasound systems used for collection included a Stork ultrasound system with an H35C convex array (Stork Healthcare Co., Ltd., China), a Mindray ultrasound system with an SC5-1 convex array (Mindray Bio-Medical Electronics Co., Ltd., China), and a Philips ultrasound system with an Epiq 7 C5-1 convex array (Philips Medical Systems, Inc., the Netherlands). In total, the three datasets included 5,704 LUS images from 108 COVID-19 patients. The 5,704 LUS images were manually labeled with different types of ultrasound images, reflecting the degree of lung involvement: A-line, A&B-line, B1-line, B2-line, B1&B2-line, and consolidation (see Figure 1). In the proposed network, Hu et al. [25] first extracted two feature maps from the LUS image: gradient field map and *K*-means clustering map. The gradient field map was highly sensitive to A-lines, and the *K*-means clustering map was highly sensitive to B-lines. The two extracted feature maps and the LUS image constituted the three channel inputs to the deep learning model ResNext [92]. A Squeeze-and- Excitation network (SE) [93] was used to generate an activation value for each channel input. For patient-based evaluation, each frame from the same patient was scored based on the scoring system proposed in another study [94], where A-line, A&B-line, B1-line, B1&B2-line, B2-line, and consolidation were scored as 0, 1, 2, 2.5, 3, and 4, respectively. The final lung involvement score for each patient was the average score of all frames obtained from that patient. Finally, for an additional set of videos acquired from 8 patients, the correlation between the score and the partial pressure of CO_2_ (pCO2), an indicator of the patient’s respiratory function, was analyzed.

Overall, the classification accuracy of the proposed model was higher than other tested models. Specifically, the diagnostic model achieved 94.39% accuracy, 82.28% precision, 76.27% sensitivity, and 96.44% specificity. Using feature maps of gradient field and *K*-means clustering increased the classification accuracy by 2.8% on average. The Pearson correlation coefficient between pCO2 and the predicted score was 0.73 (*p* < 0:001), suggesting that the proposed scoring system can help doctors evaluate the lung involvement of COVID-19 patients.

## 4. Open-Access Web Platform for Crowd-Sourcing Datasets and Benchmark Testing

Born et al. [60] introduced the POCOVID dataset, which initially included 64 lung POCUS video recordings (39 videos of COVID-19, 14 videos of typical bacterial pneumonia, and 11 videos of healthy patients) collected from several online data sources (see details in Table 2). These collected videos were each confirmed by a medical doctor to have visible COVID-19 or pneumonia disease-specific patterns. A total of 1,103 images (654 COVID-19, 277 bacterial pneumonia, and 172 healthy) were extracted from the 64 videos.

To classify COVID-19 patients from typical bacterial pneumonia or healthy patients, Born et al. [60] proposed the convolutional neural network POCOVID-Net, which was based on the VGG16 architecture [54]. POCOVID-Net was pretrained on Imagenet to extract image features such as shapes and textures. Data augmentation techniques were used to diversify the dataset and prevent overfitting. In addition to frame-based classification, Born et al. [60] also proposed classifying videos based on frame-wise scores with two methods: (1) taking a majority vote of the predicted classes and (2) selecting the class with the highest average probability.

The results reported in [60] were obtained with 5-fold cross-validation. AUC scores for classifying COVID-19, pneumonia, or healthy were ≥0.94. In particular, the AUC score of COVID-19 detection was 0.94. The image-wise sensitivity, specificity, precision, and *F*1-score for COVID-19 was 96% and 79%, 88% and 0.92, respectively. The authors suggested that the main reason for the low specificity was the small sample size of healthy images compared to COVID-19 images. For video classification, both methods achieved an accuracy of 92%. In addition to the initial collection of the dataset, Born et al. [60] also built an open-access web platform where users can contribute to the POCOVID open-access dataset by uploading their ultrasound recordings. Additional benefits of this contribution include ease of user access to the trained model to perform either a rapid screening of new data or a baseline comparison to a new network architecture, as implemented by Awasthi et al. [58].

## 5. Comparison with Other Medical Imaging Techniques

Horry et al. [61] compared the performance of deep learning models among three imaging modalities: X-ray, CT, and LUS. LUS images for COVID-19, pneumonia, and normal conditions were obtained from the publicly accessible POCOVID-Net data set [60]. COVID-19 CXRs were obtained from the publicly accessible COVID-19 image data collection [63]. For pneumonia (non-COVID-19) and normal condition X-rays, the authors used the National Institutes of Health (NIH) Chest X-Ray datasets. CT scans for COVID-19 and non-COVID-19 were obtained from the publicly accessible COVID-CT Dataset [62]. More dataset details are available in Table 2.

In total, Horry et al. [61] trained seven architectures: (1) VGG16 and VGG19 [72], (2) RESNET50 V2 [73], (3) INCEPTION V3 [95], (4) XCEPTION [96], (5) INCEPTIONRESNET V2 [97], (6) NASNETLARGE [66], and (7) DENSENET121 [98]. Each classifier was trained on the ImageNet [99] weights for transfer learning. The testing results showed that the simpler VGG classifiers were more trainable on the three imaging modalities and provided more consistent results across these three imaging modalities. By comparison, the more complex models tended to either overfit in early epochs or failed to converge, potentially due to the small data set. Based on the initial testing results, VGG19 was chosen for the multimodal image classification testing. With the selected VGG19 model, for each experiment listed in Table 4, extensive performance tuning was conducted by adjusting multiple parameters, including learning rate, batch size, node size, and drop rate. The best parameter setting for each experiment was identified after training.

Table 4 lists classification results for each experiment [61]. For experiments of classifying COVID-19 and non-COVID pneumonia versus healthy lungs, LUS provided better results than X-Ray with a sensitivity (recall) of 97% and a positive predictive value of 99%. In classifying COVID-19 versus non-COVID pneumonia, LUS similarly provided better results than X-ray with a sensitivity of 100% and a positive predictive value of 100%. CT performed the worst among three imaging modalities, with a sensitivity of 83% and a positive predictive value of 79% when classifying COVID-19 versus non-COVID-19 scans. Horry et al. [61] suggested that the poor performance of CT experiments may be due to the limited sample size and the variable quality of the COVID-19 data sets. Overall, *F*1 scores achieved in these experiments exceeded 80%. These results demonstrate that the VGG19 classifier with transfer learning has the potential to provide a fast and simple option to implement a machine learning model for multiple imaging modalities, and as a result, is a useful tool in the COVID-19 pandemic.

## 6. Summary and Outlook

In this review, we discussed nine research articles exploring the application of deep learning in ultrasound imaging of COVID-19. Overall, these research articles demonstrate that deep learning has strong potential to aid LUS diagnosis of COVID-19. The applications of deep learning in LUS diagnosis of COVID-19 include distinguishing COVID-19 patients from non-COVID-19 pneumonia patients or healthy patients [54–61], evaluating the severity of lung involvement of COVID-19 patients [25, 59], and assessing the quality of LUS images of COVID-19 patients [56]. Regarding the specific deep learning architecture implemented, six studies used “out-of-the-box” architectures as backbones [54, 55, 57, 58, 60], while the remaining three proposed new architectures [25, 56, 59] with the aims of improving the robustness of predictions and learning more distinctive features of input images. When exploring model explainability for both new and pretrained networks, CAMs are commonly applied to visualize discriminative image regions for a specific category [54, 56, 57]. Based on the frame-based classifier, four of the studies further built video-based or patient-based classifiers, which are more desirable in clinical settings [54, 57, 59, 60].

Because ultrasound examination of COVID-19 patients is less established, fewer COVID-19 LUS datasets were available in comparison to other imaging modalities such as CT and X-ray. Availability may also be reduced by the greater flexibility in LUS image acquisitions in comparison to CT and X-ray. Nonetheless, deep learning classifiers for LUS images achieved better performance than those of other imaging modalities, including CT and X-ray [61]. When comparing deep learning performance to human predictions, the deep learning models achieved better results when distinguishing COVID-19 patients from non-COVID-19 pneumonia patients or HPE patients [57]. Due to the scarcity of LUS images of COVID-19 patients, most studies used data augmentation techniques to diversify datasets [25, 54, 57, 59–61].

**Figure 7:**
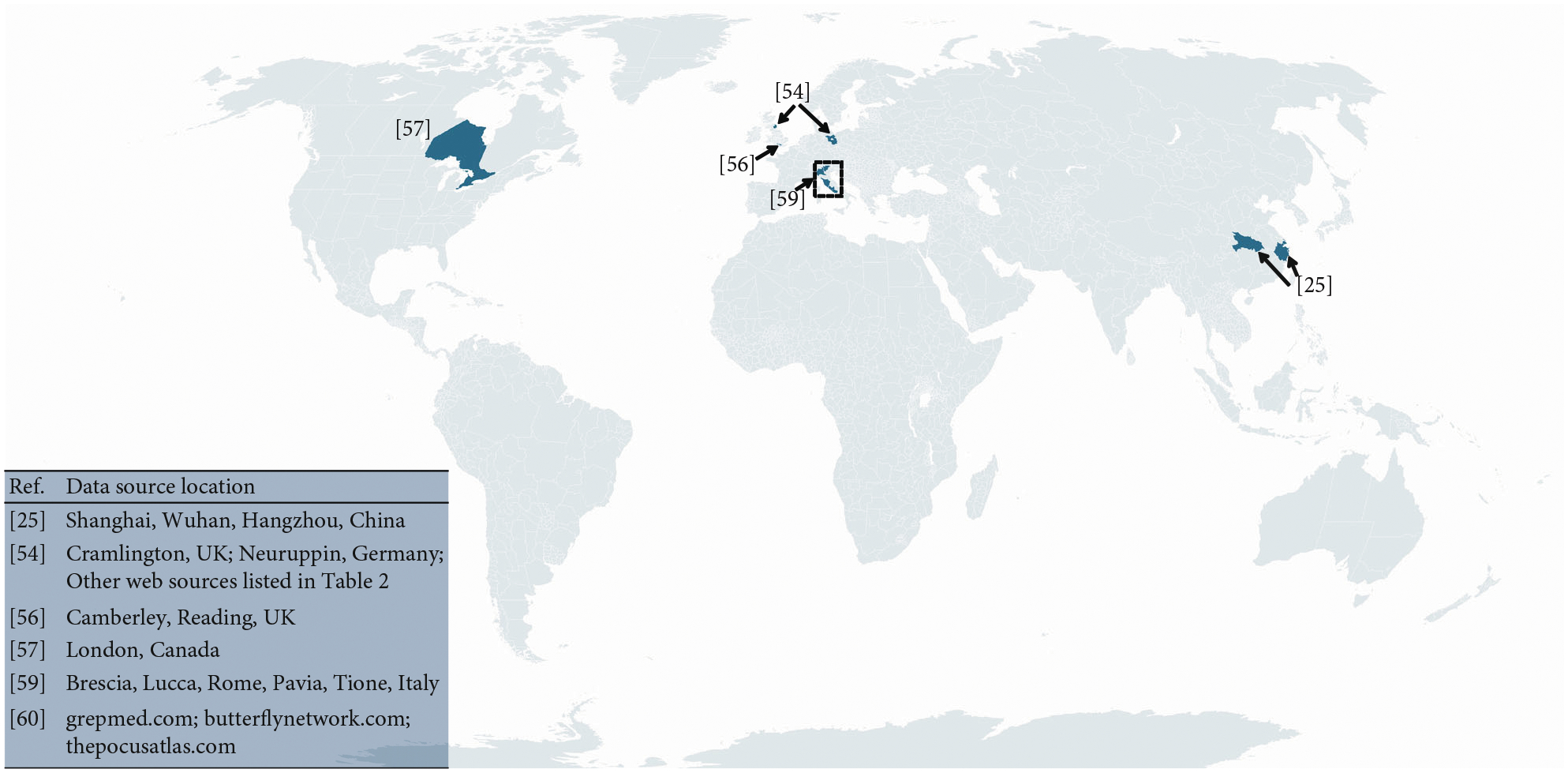
Graphical location summary of data sources described throughout this article.

The locations of data sources for the summarized studies include five countries (i.e., China, Germany, United Kingdom, Italy, and Canada) and multiple online platforms that accept LUS image uploads worldwide. These locations are displayed in Figure 7, representing a total of no more than 400 patients with COVID-19 positive ultrasound images or videos. In addition, a LUS deep learning dataset from Shenzhen, China, was also compiled, containing 678 videos from 71 COVID-19 patients [100]. Given that COVID-19 has caused more than 200 million infected cases, it is clear from Figure 7 that there are gaps in locations and patient numbers. Filling these gaps will help to ensure that the LUS deep learning community produces truly global solutions to our global pandemic.

Despite its promise, there are three immediate limitations of deep learning applications to aid LUS diagnosis of COVID-19. First, the usage of ultrasound imaging equipment can be highly operator dependent, which may cause inconsistency of training and testing results for deep learning models. In the future, this limitation may be addressed with robotic approaches. Second, to train a robust and generalizable deep learning model, larger datasets with appropriately balanced distributions of patient locations, ultrasound system manufacturers, image acquisition settings, and consistent labels are necessary. Incorporating raw channel data, which is less sensitive than B-mode images to some system settings, may also assist with improving model generalization. Third, although some studies used Grad-CAM to visualize the learned features of deep learning models, these explainability characterizations were not consistent across datasets. Ultimately, more studies are needed to address the interpretability and trustworthiness of deep learning models.

As explorations of the role of deep learning in LUS for COVID-19 patients are still underway (e.g., [101–104]), we believe that in the near future, more research implementing deep learning applications for ultrasound imaging of COVID-19 will be available. These future studies, in combination with the pioneering studies described herein, are expected to provide impactful point-of-care solutions to combat the COVID-19 pandemic. The totality of these studies is also expected to provide useful benchmarks and implications for possible future outbreaks that involve respiratory disease and mutations of SARS viruses.

## Figures and Tables

**Table 1:** Number of example images for each deep learning approach summarized in this review.

Study	Total number of examples	Training/validation/testing split
Born et al. [54]	3,234 images (from 179 videos and 53 still images not associated with videos)	5-fold cross validation
Roberts and Tsiligkaridis [55]	3,119 images (from 195 ultrasound videos)	5-fold cross validation
Baum et al. [56]	42,427 images (from 49 patients)	5-fold cross validation on 28,122 images (diagnosis assistance module) 34%/0%/66% (quality assessment module)
Arntfield et al. [57]	121,381 images (from 612 videos of 243 patients)	82%/8%/10%
Awasthi et al. [58]	1,137 images (from 64 videos)	5-fold cross validation
Roy et al. [59]	58,924 frames (from 277 videos of 35 patients)	78%/0%/22% (frame-based predictor) 5-fold cross validation on 60 videos (video-based predictor)
Hu et al. [25]	5,704 images (from 108 patients)	67%/0%/33%
Born et al. [60]	1,103 images (from 64 videos)	5-fold cross validation
Horry et al. [61]	1,103 ultrasound images 746 CT image slices 60,798 X-ray scans	80%/0%/20%

**Table 2:** Studies, associated datasets, and data sources summarized in this review.

Study	Dataset	Data source
Born et al. [54]	Updated POCOVID dataset (December 2020) [54]: 179 videos and 53 still images not associated with videos, 4 classes of data (i.e., COVID-19, bacterial pneumonia, non-COVID-19 viral pneumonia, and healthy controls)	The Northumbria Healthcase NHS foundation trust Medizinische Hochschule Brandenburg Theodor Fontane https://www.grepmed.com/ https://www.butterflynetwork.com/ https://www.thepocusatlas.com/ https://litfl.com/lung-ultrasound-covid-19-cases https://www.stemlynsblog.org/ https://clarius.com/ https://everydayultrasound.com/ https://radiopaedia.org/ http://www.acutemedicine.org/defaultsite https://www.bcpocus.ca/ https://www.youtube.com/ https://sonographiebilder.de/sonographie-atlas LUS videos and images retrieved From publications [54]
Roberts and Tsiligkaridis [55]	Updated POCOVID dataset (Nov 2020) [60]: 3,119 frames from 195 ultrasound videos	https://www.grepmed.com/ https://www.thepocusatlas.com/ https://www.butterflynetwork.com/ https://radiopaedia.org/
Baum et al. [56]	25,800 LUS images from 37 COVID-19 positive patients and 16,627 LUS images from 12 COVID-19 negative patients. Image quality was manually labeled as sufficient (*n* = 41490) or insufficient (*n* = 937).	All images were obtained in 2 hospitals in the UK.
Arntfield et al. [57]	121,381 LUS images sampled from 612 LUS examination videos of 243 patients (81 hydrostatic pulmonary edema (HPE), 78 non-COVID-19, and 84 COVID-19)	Datasets were collected within 2 tertiary hospitals of London Health Sciences Centre (Canada).
Awasthi et al. [58]	POCOVID dataset [60]: 1,137 images (678 COVID-19, 277 backterial pneumonia and 182 healthy controls) sampled from 64 videos	POCOVID dataset [60]
Roy et al. [59]	Italian COVID-19 lung ultrasound database (ICLUS-DB) [59]: 58,924 frames (277 LUS videos) from 35 patients (17 COVID-19, 4 COVID-19 suspected, 14 healthy and symptomless individuals). All frames were labeled with four COVID-19 severity levels (0 to 3). 60 videos across all 35 patients were annotated at video-level. 1,431 frames were semantically annotated at a pixel-level.	The data were acquired within 5 different clinical centers in Italy.
Hu et al. [25]	5,704 LUS images from 108 COVID-19 patients. All images were manually labeled with different degrees of lung involvement: A-line, A&B-line, B1-line, B2-line, B1&B2-line, and consolidation.	Datasets were obtained from four medical centers in China.
Born et al. [60]	Initial POCOVID dataset (May 2020) [60]: 1103 images (654 COVID-19, 277 bacterial pneumonia and 172 healthy controls) sampled from 64 videos	https://www.grepmed.com/ https://www.butterflynetwork.com/index.html https://www.thepocusatlas.com/
Horry et al. [61]	Ultrasound: 1,103 LUS images (654 COVID-19, 277 non-COVID-19 pneumonia, 172 no finding) from POCOVID-net dataset [60] CT: 746 CT image slices (349 COVID-19 and 397 non-COVID-19 pneumonia) from COVID-CT dataset [62] X-ray: 115 X-ray scans of COVID-19 patients from COVID-19 image data collection [63], 322 XRay scans of non-COVID-19 pneumonia patients, and 60,361 X-ray scans with no finding from NIH chest X-ray dataset [64] (all the above datasets were based on downloads made on May 11, 2020 [61])	POCOVID-net dataset [60] COVID-CT dataset [62] COVID-19 image data collection [63] NIH chest X-ray dataset [64]

**Table 3:** Definition of performance metrics reported for each reviewed article, where TP: true positive; TN: true negative; FP: false positive; FN: false negative.

Study	Definition of performance metrics
[25, 54–56, 58, 60, 61]	Accuracy = (TP + TN)/allevaluatedcases
[25, 58–61]	Sensitivity (or recall) = TP/(TP + FN)
[25, 58, 60]	Specificity = TN/(TN + FP)
[58–61]	Precision = TP/(TP + FP) *F*1 score = 2(sensitivity × precision)/(sensitivity + precision)
Born et al. [54]	TP: number of cases correctly identified as COVID-19 TN: Number of cases correctly identified as healthy or pneumonia
Roberts et al. [55]	TP: number of cases correctly identified as COVID-19 TN: number of cases correctly identified as non-COVID-19 (including both healthy and pneumonia cases)
Baum et al. [56]	For quality assessment models: TP: number of cases correctly identified as sufficient TN: number of cases correctly identified as insufficient For diagnostic assistance model: TP: number of cases correctly identified as COVID-19 TN: the number of cases correctly identified as non-COVID-19
Arntfield et al. [57]	AUC (COVID-19): the AUC for differentiating COVID-19 cases from non-COVID-19 pneumonia or HPE cases AUC (NCOVID): the AUC for differentiating non-COVID-19 pneumonia cases from COVID-19 or HPE cases. AUC (HPE): the AUC for differentiating HPE cases from COVID-19 or non-COVID-19 pneumonia cases.
Awasthi et al. [58] Born et al. [60]	TP: number of cases correctly identified as COVID-19 TN: number of cases correctly identified as healthy or pneumonia in the definition of accuracy and number of cases correctly identified as non-COVID-19 in the definition of COVID-19 sensitivity, COVID-19 specificity, COVID-19 precision FP: number of cases wrongly identified as COVID-19 FN: number of cases wrongly identified as non-COVID-19
Roy et al. [59]	TP: number of cases predicted successfully to have certain severity score FP: number of cases predicted wrongly to have that score FN: number of cases predicted wrongly to not have certain score
Hu et al. [25]	TP: number of cases predicted successfully to have certain pathologic feature (i.e., A-line, A&B-line, B1-line, B1&B2-line, B2-line, and consolidation) FP: number of cases predicted wrongly to have certain pathologic feature TN: number of cases predicted successfully to not have certain pathologic feature FN: number of cases predicted wrongly to not have certain pathologic feature
Born et al. [60]	AUC (COVID-19): the AUC for differentiating COVID-19 cases from pneumonia or healthy cases
Horry et al. [61]	For “Normal vs. COVID-19 and pneumonia” studies: TP: number of cases correctly identified as COVID-19 or pneumonia FP: number of cases wrongly identified as COVID-19 or pneumonia FN: number of cases wrongly identified as normal For “COVID-19 vs. pneumonia” studies: TP: number of cases correctly identified as COVID-19 FP: number of cases wrongly identified as COVID-19 FN: number of cases wrongly identified as pneumonia For “COVID-19 vs. non COVID-19” studies: TP: number of cases correctly identified as COVID-19 FP: number of cases wrongly identified as COVID-19 FN: number of cases wrongly identified as non COVID-19

**Table 4:** Summary of multimodality experiments and results obtained with VGG19 [61], where *P* is positive predictive value, *R* is recall rate (also known as sensitivity), and *F*1 is *F*1 score.

Imaging modality	Experiment	Classification	Results
X-ray	COVID-19 and pneumonia vs. normal	COVID-19 + pneumonia	*P*: 0.85 *R*: 0.83 *F*1: 0.84
		Normal	*P*: 0.86 *R*: 0.88 *F*1: 0.87
Ultrasound	COVID-19 and pneumonia vs. normal	COVID-19 + pneumonia	*P*: 0.99 *R*: 0.97 *F*1: 0.98
		Normal	*P*: 0.94 *R*: 0.98 *F*1: 0.96
X-ray	COVID-19 vs. pneumonia	COVID-19	*P*: 0.86 *R*: 0.86 *F*1: 0.86
		Pneumonia	*P*: 0.89 *R*: 0.89 *F*1: 0.89
Ultrasound	COVID-19 vs. pneumonia	COVID-19	*P*: 1.00 *R*: 1.00 *F*1: 1.00
		Pneumonia	*P*: 1.00 *R*: 1.00 *F*1: 1.00
CT	COVID-19 vs. non-COVID-19	COVID-19	*P*: 0.79 *R*: 0.83 *F*1: 0.81
		Non-COVID-19	*P*: 0.84 *R*: 0.81 *F*1: 0.83

## Data Availability

No new data were created for this manuscript.
